# Improving the quality of care in nursing home organizations with urgent quality issues: design and effectiveness of a Dutch government-funded support programme

**DOI:** 10.1186/s12913-023-09538-w

**Published:** 2023-05-18

**Authors:** Paulien Vermunt, Yael Reijmer, Clariska van Biessum, Valerie de Groot, Bellis van den Berg, Henk Nies

**Affiliations:** 1 Vilans, the National Centre of Expertise for Long-Term Care, PO Box 8228, 3503 RE Utrecht, the Netherlands; 2 Faculty of Social Sciences, Department of Organization Sciences, VU University Amsterdam, De Boelelaan 1105, 1081 HV Amsterdam, the Netherlands

**Keywords:** Person-centred care, Resident safety, Nursing home organizations, Quality improvement

## Abstract

**Background:**

Various societal developments are currently challenging the ability of European nursing home organizations to meet quality standards. To support nursing home organizations throughout the Netherlands in quality improvement (QI), the Dutch government launched a nationwide programme in 2016 entitled ‘Dignity and pride’ (D&p). As part of this programme, participating nursing home organizations followed a tailored trajectory centred around intensive, on-site support from external expert coaches. For this study, we evaluated to what extent quality improvements were realized in the programme, paying particular attention to the role of the expert coaches.

**Methods:**

Thirty-six nursing home organizations were included. At the start of D&p, the majority of these organizations (78%) had major quality issues as determined by the Health Care Inspectorate. Information on quality of care at the start versus end of the programme was obtained from improvement plans and final evaluation reports. Quality of person-centred care (PCC) and resident safety were quantified using a standardized assessment tool based on national guidelines, with improvements analysed using two-sided paired-sample T-tests. In addition, semi-structured interviews were conducted with 14 coaches and 29 healthcare professionals, focusing on the greatest benefits of programme participation and on the added value of the expert coaches.

**Results:**

After completion of the programme, 60% of the organizations scored a 4 (= good) on PCC and resident safety, and none scored a 2 or less (average improvement on a 5-point scale for both themes: 1.9 points, *p* < 0.001). Interviewees confirmed that the quality of care had both improved and become more person-centred. The expert coaches were credited with substantially contributing to the QI process by offering an outsider’s perspective, bringing in experience and expertise, and helping the organization stay committed and focused.

**Conclusions:**

Our study results suggest that the D&p programme was associated with improved quality of care in nursing home organizations with urgent quality issues. However, offering on-site tailored support through a nationally coordinated, government-funded programme is both time- and labour-intensive, and therefore not feasible in every healthcare setting. Nevertheless, the findings provide valuable insights for future QI support strategies.

## Background

Throughout Europe, many nursing home organizations are currently struggling to provide and maintain a high quality of care in the face of challenging societal developments [1, 2]. Among these developments are increasing numbers of older people with complex care needs [1, 3], a consequence of population ageing [4], and a historic shortage of skilled nursing-home personnel [5]. In the Netherlands, the above challenges have resulted in a relatively high number of nursing home organizations with severe quality issues, as observed by the Dutch Healthcare Inspectorate in 2014 [6]. In these organizations, quality of care deficiencies were found to seriously compromise the safety and well-being of residents, generating urgent calls for national measures aimed at quality improvement (QI) in long-term care organizations for older people [1, 2].

A common strategy for stimulating QI is to define standards for quality care and to hold care home organizations accountable for meeting these standards [1, 7]. Incorporating such quality standards into everyday practice, however, is far from straightforward, as each organization must adapt QI interventions to their own local context and culture [2, 8, 9]. Therefore, in 2016, to facilitate implementation of the national Quality Framework for Nursing Home Care, as issued by the Dutch Health Care Institute [6, 10], the Dutch Ministry of Health, Welfare and Sport launched a nationwide government-funded support programme for the nursing home sector. Named ‘Dignity and pride’ (D&p) [6, 11], the programme aimed both to contribute to a dignified life for older people living in nursing homes and to help healthcare professionals take more pride in their work. The ministry commissioned Vilans, the National Centre of Expertise for Long-term Care in the Netherlands, to execute the programme.

**Fig. 1:**
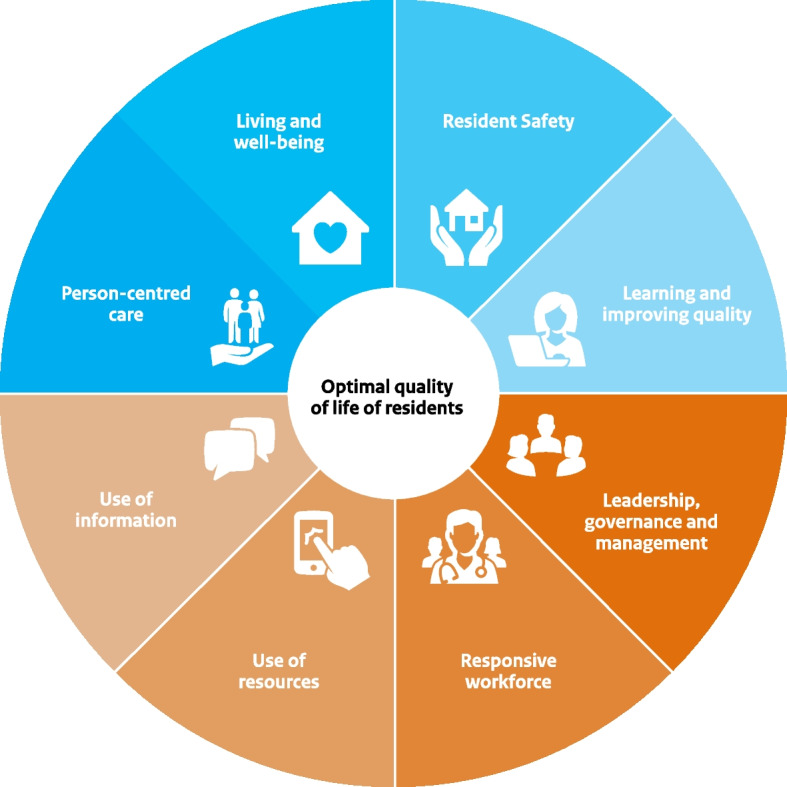
The Dutch quality framework for nursing home care

The design of D&p was based on the experience of previous Dutch programmes in long-term care [12–14]. Following a whole-system approach, D&p targeted all aspects of quality care—including conditional factors such as strategic personnel planning and governance [10, 15]; Fig. 1)—while also involving all relevant stakeholders—including the board and management, healthcare staff, supporting staff, and residents and their informal caregivers [11]. Helping to embed complex QI interventions into the routine of nursing home care [9], this whole-system approach is thought to be a prerequisite for successful and sustainable change [8, 16].

**Fig. 2:**
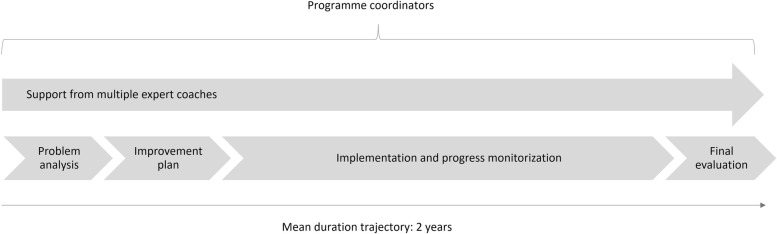
Design of D&p improvement trajectories

The D&p programme consisted of two main elements, the first of which entailed the provision of on-site tailored support by external expert coaches (sometimes referred to as ‘facilitators’ or ‘change agents’) whose task was to guide the organizations through each step of the improvement trajectory (Fig. 2). By partnering both with the board and management, and with on-site champions and nurses in care teams, an important aim of the expert coaches was to promote ownership and capacity building within each organization. Such ‘blended facilitation’ combines the advantages of an outsider’s perspective with an internal driving force [17]. As the programme’s second main element, participating organizations were encouraged to share their ‘lessons learned’ through the programme website, newsletters, theme-specific meetings and an annual conference. This ‘knowledge component’ was open to all Dutch nursing home organizations, even those not participating in D&p.

In recent years, various nationwide support programmes for nursing home care have been initiated throughout Europe [18–22]. Although all of these programmes have been designed to facilitate QI, D&p has proven fairly unique not only in terms of its ability to simultaneously address a variety of quality themes, but also due to the relatively long and intensive on-site support provided by the external expert coaches. In this article, we investigate to what extent quality improvements were realized in the participating nursing home organizations, using the concepts of ‘person-centred care’ (PCC) and ‘resident safety’ as indicators for quality of care. In addition, we elaborate on the added value of the external expert coaches within D&p, whose contributions have often been viewed as a powerful component of QI strategies [8, 17].

## Methods

### Enrolment in the D&p programme

The D&p programme was open to all nursing home organizations with urgent quality issues as determined by the Dutch Health Care Inspectorate (HCI) or by the executive board of a nursing home organization. In the Netherlands, the HCI supervises healthcare services, including nursing home organizations, to make sure that these comply with the relevant legal and regulatory requirements. As a supervisory activity, the HCI pays unannounced visits to care organizations to observe daily practice and to interview healthcare professionals about their routines and standards. In nursing home care, the national Quality Framework for Nursing Home Care [6, 10] hereby serves as the standard for high quality care. Findings are then summarized in a report assigning a score from 1 (inadequate) to 5 (excellent) to the themes and tenets of the Quality Framework, amongst which PCC and resident safety. Based on the report the HCI can label the quality of care in a nursing home organization as ‘insufficient’, reflecting urgent quality issues. If quality of care deficiencies are thought to seriously compromise the safety and well-being of residents, organizations are placed under strict supervision of the HCI and summoned to implement drastic changes to avoid closure. On request of the Dutch Ministry of Health, Welfare and Sport, the HCI actively encouraged both nursing home organizations with insufficient quality of care and organizations under strict supervision to participate in the D&p programme. In addition, organizations could also independently of the HCI register to the programme if the executive board felt that help was needed to overcome urgent quality issues and comply to the standards of the Quality Framework.

### Nursing home organization selection

In total, 65 organizations were enrolled in the D&p programme between 2016 and 2019. Only those organizations that had completed the improvement trajectory were included in our study, so that information on quality of PCC and resident safety at both the start and the end of the trajectory was available. At the start of our study in February 2020 (reference date), 38 of the 56 nursing home organizations that had already completed the trajectory were selected based on their date of completion being no longer than 1.5 years prior (between June 2018 and February 2020). This period was chosen to minimize information bias and to increase the chance that healthcare professionals involved in the programme would still be working at the nursing home organization. Two organizations were excluded for incomplete documentation, leaving 36 nursing home organizations for inclusion in this study. Participating nursing home organizations were located throughout the Netherlands and all provided care to older people.

### Nursing home organization characteristics and context

The main characteristics of the 36 nursing home organizations are given in Table 1. In the Netherlands, nursing home organizations usually consist of several individual nursing homes at different locations, also referred to as ‘facilities’. The 36 nursing home organizations participating in our study varied in the number of facilities constituting the organization (1 to 30 facilities, with a total of 281 facilities). Furthermore, participating nursing home organizations mostly held hundreds to thousands of residents, and a large number of professional healthcare employees, including (para)medical, auxiliary, and psychosocial staff. Slightly more than half of the nursing home organizations were located in the western urban areas of the Netherlands, with the rest more rurally located. The western urban areas cover the provinces of North-Holland, South-Holland, Flevoland and Utrecht and are characterized by several agglomerates of large cities, concentrated industry and a high population density. In these areas, together also called ‘Randstad’, the shortage of skilled nursing-home personnel is higher than in the rural areas, while the number of older people in need of nursing home care is equally increasing. Twenty-eight of the nursing home organizations in our study showed an insufficient quality of care as determined by the HCI; Sixteen of the organizations were under strict supervision of the HCI before the start of the programme.

**Table 1:** Characteristics of included nursing home organizations (*N* = 36)

Mean duration of the trajectory	2 years
Number of facilities within the nursing home organization	
*1–2 facilities*	14 (39%)
*3–10 facilities*	10 (28%)
> *10 facilities*	12 (33%)
Average number of healthcare professionals per organization^a^	812 (32–3232)
Average number of residents per organization	367 (29–2400)
Location of the nursing home organization	
*Urban area*	20 (55%)
*Rural area*	16 (45%)
Insufficient quality of care according to HCI	28 (78%)
Under strict supervision of the HCI	16 (44%)

Mean ± SD or n (%) are given

*HCI* Health Care Inspectorate

^a^including the board and management, (para)medical, auxiliary and psychosocial staff, excluding support staff such as administrative, facility services, housekeeping, kitchen personnel etc.

Participating nursing home organizations were often facing troubling issues at all levels of their organization, posing a challenge to the provision of PCC and frequently resulting in serious safety risks for residents. Common examples of such issues included frequent changes of the board and management over a short period of time, radical reorganizations involving major changes in tasks and responsibilities, and severe financial problems. Almost all participating organizations had to deal with a lack of (skilled) personnel, a high absenteeism rate and a high percentage of temporary workers.

### Text box. Design of the D&p improvement trajectories

The mean duration of a single improvement trajectory was 2 years. Participation was free of charge, but nursing home organizations were obliged to pay back all expenses if they failed to adhere to the programme agreement and/or withdrew from the support programme without sufficient reason.

Specialized in change management and / or the practical operationalization of the programme’s quality standards, multiple external coaches were hired to provide on-site support to nursing home organizations on both strategic and operational levels. Coaches were involved in all stages of the trajectory, including (Fig. 2):

- *Problem analysis and improvement planning:* Based on audit reports, reports from the Health Care Inspectorate, research on residents and informal care experiences, interviews with stakeholders, and care observations, each improvement trajectory started with a structured analysis of the major quality issues within an organization. Rooted in this analysis, a tailored action plan would then be developed to address the specific needs of the organization. In line with the whole-system approach of D&p, action plans were required to cover all eight ‘quality themes’ of the national Quality Framework for Nursing Home Care [10, 15] (Fig. 1). Each plan was co-designed and approved by several stakeholders, including the organizations’ various boards (executive, supervisory, and the employees’ and clients’ advisory board).
- *Implementation:* In addition to being tailored to each organization’s specific needs, interventions described in the action plan were deliberately embedded in the routines of nursing home care. Although exact intervention strategies varied across trajectories, they generally covered four stages of change, following the model of ‘intervention mapping’ [23]: 1) gaining insight into the current situation; 2) envisioning the desired situation and setting goals accordingly; 3) translating the derived vision and goals into an operational plan; and 4) implementing change and reflecting on the results. The four stages of intervention mapping together constitute a full PDCA-cycle (shorthand for a ‘plan-do-check-act’ methodology). With respect to PCC and resident safety, frequently applied interventions included client evaluations and complaint analysis (stage 1), vision dialogue sessions and team action planning (stage 2), appointing specialist nurses and redesigning work processes (stage 3), and on-the-job coaching and casuistry discussion in team meetings (reflecting on situations in daily practice to identify functional or dysfunctional behaviours and procedures (stage 4)).
- *Progress monitorization and final evaluation*: Continuously monitored by the expert coaches and a D&p programme coordinator, each organization’s implementation strategy was adapted as needed throughout the process. To this purpose, interventions in all four stages of intervention mapping were regularly evaluated, following the PDCA-cycle. To assist them in continuing to monitor and improve their organization’s quality of care even after completion of the programme, managers and certified (assistant) nurses were also trained by the expert coaches to work according to the PDCA cycle. At the end of each organization’s trajectory, a final evaluation involving the organization’s employees, the coaches and a programme coordinator was used to assess the organization’s progress and crystalize the ‘lessons learned’ throughout the programme.

### Study design

Using a mixed quantitative and qualitative retrospective study design, the quality of both PCC and resident safety in participating nursing home organizations were quantified using a standardized assessment tool (see passage ‘scoring procedure’ below). Information was gathered from programme-related documentation and external quality reports, and from semi-structured interviews conducted by the authors with participating organization staff (*n* = 39), expert coaches (*n* = 14) and programme coordinators (*n* = 3), to gain a more in-depth understanding of the programme’s trajectory. Specific details regarding the quantitative and qualitative methods are provided below. Our study design was retrospective as it was based on existing documentation that was gathered in the past (improvement plans and final evaluation reports). Because we included only those organizations that had completed the improvement trajectory, we were able to compare the quality of PCC and resident safety between the start and the end of the trajectory. Furthermore, even though interview-data was collected for the purpose of this study, interviewees were asked to recall past experiences on an outcome of interest that had already occurred.

### Quantitative evaluation of quality of care

Quantitative data on each participating nursing home organizations’ quality of care at the start versus the end of the improvement trajectory were obtained from obligatory, standardized problem analyses and improvement plans, and from final evaluation reports, both of which included a thorough description of the quality of care on all eight quality themes. Given the immediate relevance for residents and their relatives of *PCC*—representing the ‘soft side’ of care (interaction, relationships)—and *resident safety*—representing the ‘hard side’ of care (protocols, standards)—we chose to focus on these two themes specifically as indicators of quality care. Reports from the Dutch Health Care Inspectorate made within 3 months prior to, or post, the start and end dates of the trajectory were also used for qualitative care assessments.

#### Scoring procedure

Three of the authors (PV, YR, CvB) analysed the above-mentioned documentation of each nursing home organization, taking care to select all information related to PCC and resident safety. A standardized scoring form was developed for this purpose, with eight operational tenets per theme as described by the Quality Framework for Nursing Home Care (Table 2). For each tenet within the themes of PCC and resident safety, a pair of scores was assigned based on the information gathered in the scoring form, one per start date, one per end date. Scores ranged from 1 (inadequate) to 5 (excellent), following the scoring procedures of the Dutch Health Care Inspectorate (HCI). For each nursing home organization and for each time-point (start date or end date) two raters independently of each other analysed the available documentation and assigned scores. Time-points (start date or end date) were scored in randomized order and by different rating partners to avoid learning-effects.

**Table 2:** Eight operational tenets of PCC and resident safety as described in the Dutch quality framework for nursing home care

**Person-centred care**
1. Healthcare workers approach residents with kindness, consideration and respect
2. Care & well-being plan: created with the involvement of residents and their informal care givers carecarecaregivers
3. Care & well-being plan: drafted within six weeks
4. Care & well-being plan: based on familiarity with residents (wants, needs, preferences)
5. Care is provided in accordance with care & well-being plan
6. Multidisciplinary collaboration
7. Evaluation of care & well-being plan (together with residents/informal caregivers)
8. Needs and desires of the resident form the basis of care
**Resident safety**
1. Agreements around safe, responsible care provision and mutual learning
2. Health risks to residents are identified and made explicit
3. Resident Incident Reports (RIR) and Employee Incident Reports (EIR)
4. Follow-up on IR/EIR
5. Employees trained and competent vis-à-vis restricted procedures
6. Policies for medication safety and administration
7. Care and Constraint: reduced use of freedom-restricting measures
8. Hygienic work practices and infection prevention

The selected information and tenet scores were then compared and discussed in consensus meetings with all three researchers. When tenets were scored differently, and no consensus could be reached, a final score was determined by the third researcher based on the argumentation of the two raters.

When information was insufficient to assign a score, the tenet was left open. The ‘total scores’ for PCC and resident safety were calculated by averaging the tenet scores, resulting per nursing home organization in two total scores per theme (one per start date, one per end date). A minimum of four of the eight tenets had to be scored in order to calculate a theme’s total score, with no correlation found between the number of missing items and a theme’s total score (Spearman correlation *p* > 0.10).

#### Quantitative data analysis

Quantitative data analysis was performed using the statistical programme SPSS (version 16, SPSS Inc, Chicago, IL, USA). Changes in quality scores between the start and end of the trajectory were evaluated with a paired-sample T-test (two-sided). To evaluate potential rater bias, we compared the scores assigned by one rater to the scores assigned by the other two raters with an independent-samples T-test. None of the raters appeared to be consistently more positive or negative in assigning quality scores (*p* > 0.10).

### Interviews with stakeholders

In order to collect in-depth information about the improvement trajectories, semi-structured interviews were conducted with 12 of the 36 nursing home organizations—purposively sampled to include various residence sizes and locations within the Netherlands. Likewise, at our request, a variety of employees were invited by either the nursing home director or former D&p project leader to participate in the interviews, allowing us to obtain information from different perspectives.. All participants voluntarily participated in the interviews.

In total, 6 directors, 16 policy officers (team managers, project leaders, and quality officers) 7 certified (assistant) nurses, 14 expert coaches (strategic and operational) and 3 programme coordinators were interviewed for this study. Adapted for use with the different target groups, the topic lists included questions focused both on the greatest benefits of participation for interviewees’ daily practice and on the added value of D&p’s various design elements (Fig. 2). Specific attention was given to the role of the expert coaches, who had been intensively engaged in all stages of the improvement trajectories.

#### Qualitative data analysis

To conduct an inductive thematic analysis of the interviews [24], two of the authors (PV and VdG) separately coded the transcripts using MaxQDA 10: after selecting and coding the text fragments related to the ‘greatest benefits’ and ‘expert coaches’, the two researchers compared their identified codes and discussed them with a third researcher (YR) until consensus was reached about the relevance and meaning of the codes. Next, the codes were iteratively reviewed for coherence, refined, recoded, and clustered to identify overarching themes (axial coding).

### Ethical considerations and transparency

To ensure the independent position of the researchers and to promote transparency around the methodology used for scoring the tenets, an audit of the research was conducted by two external, independent auditors. The auditors, who were given access to the raw data and scoring forms after signing a confidentiality agreement, agreed with the assessment procedure and the findings as presented in this study.

## Results

### Improvements in quality of care

**Fig. 3:**
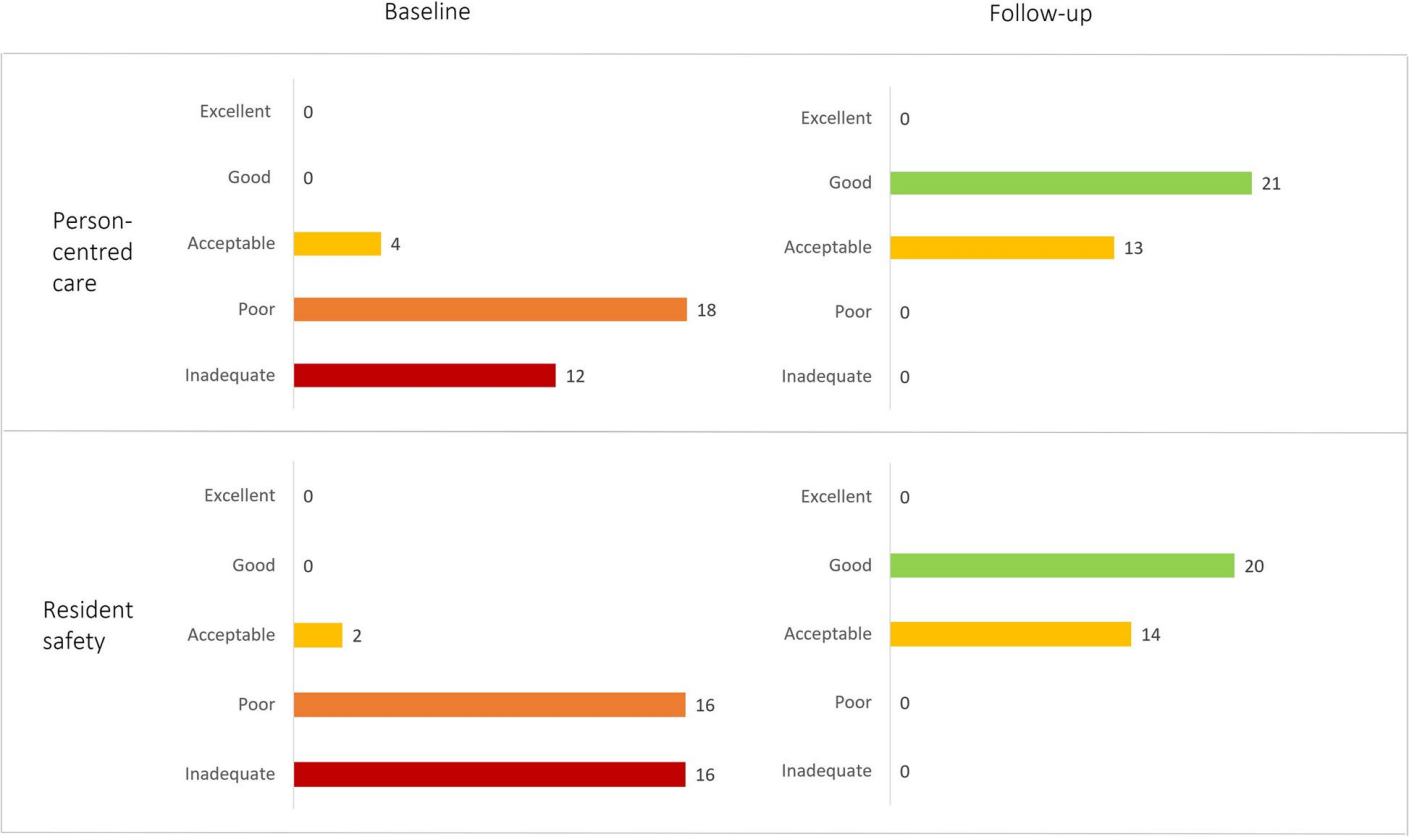
Distribution of quality scores for PCC (top) and resident safety (bottom) at the start (baseline) and end (follow-up) of the improvement trajectories

**Fig. 4:**
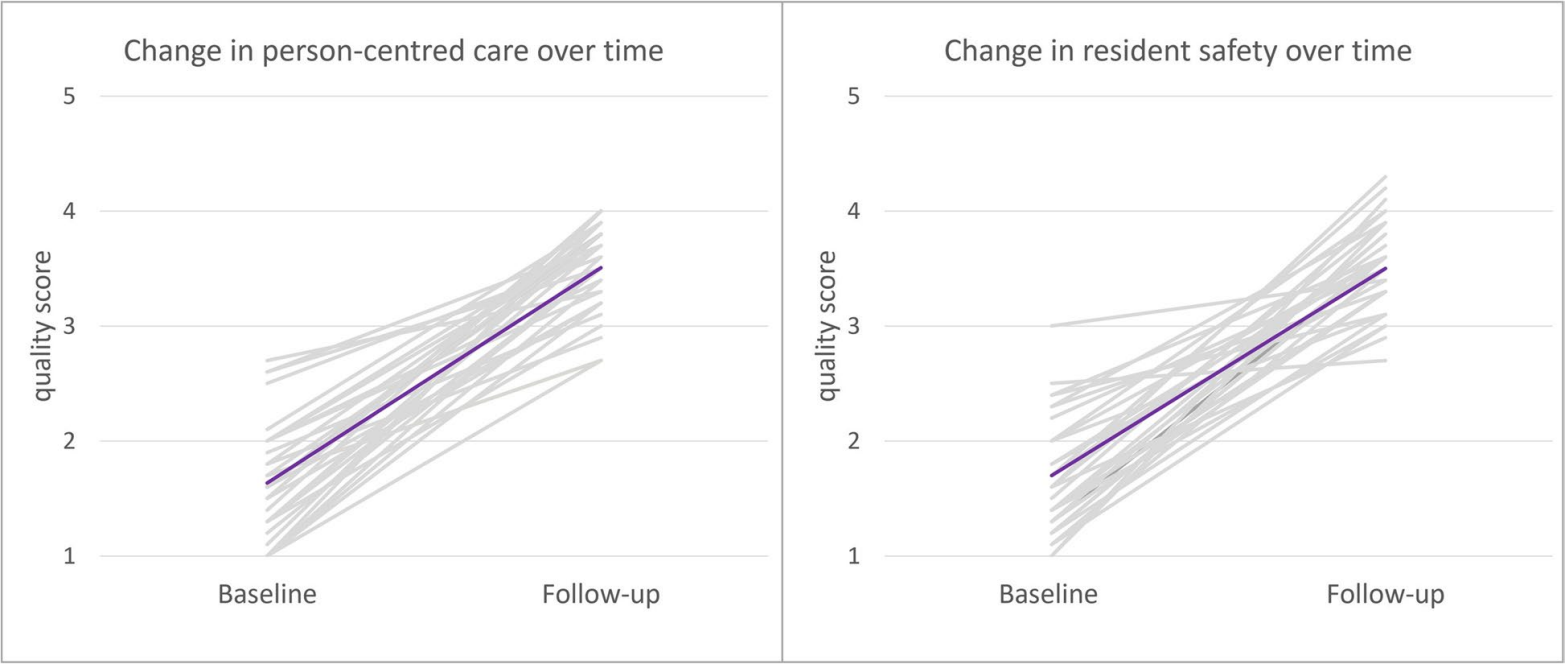
Change in quality scores for PCC and resident safety between the start and end of the improvement trajectory for each nursing home organization (light grey) and the group average (purple)

At the start of the D&p programme, 90% of the nursing home organizations studied received a score of 2 or less on PCC and/or resident safety (on a scale of 1 to 5), indicating that the quality of care failed to meet current standards. At the end of the programme, 60% of the same nursing home organizations scored a 4, indicating a good quality of PCC and resident safety, and none scored a 2 or less (Fig. 3). Nearly all of the nursing home organizations showed an improvement in PCC and resident safety following completion of the programme (average improvement was 1.9 points, *p* < 0.001; Fig. 4).

Post-hoc analysis on the eight tenets of both PCC and resident safety (Table 2) revealed no significant difference in the degree of change in scores across individual tenets (average change + 1.8–1.9 points), indicating that the improvement of PCC and resident safety was due to increased scores for all tenets. Only for the PCC tenet ‘care and well-being plan: drafted within 6 weeks’ we were unable to calculate a change in quality scores due to insufficient data at both the start and end of the trajectories.

### Greatest benefits for daily practice

In line with the outcomes of our quantitative analysis, both the healthcare professionals and expert coaches we interviewed confirmed that the quality of care had improved within the participating nursing home organizations. When asked to articulate how their organization had most benefitted from the support programme, interviewees most frequently mentioned:

1. *Improved awareness of quality standards* both in general (as defined in the Quality Framework for Nursing Home Care) and of the extent to which the organization met these standards. The effectiveness of the initial problem analysis—the first stage of D&p—was often underscored, having helped the organization gain insight into its strengths and weaknesses, and forming a starting point for a structured and focused improvement plan.
  Quality officer: ‘We all thought we were doing well with respect to person-centred care, but D&p showed us we could in fact do better.’
  Expert strategic coach: ‘The organization made a switch from unconsciously incompetent to consciously incompetent. They hadn’t been aware of what was going wrong, so they just assumed everything was going well.’

2) *An increased focus on systematic monitoring and improving quality of care* (i.e., using the PDCA cycle). Healthcare professionals emphasized the substantial impact of this systematic approach within their organization: directors, policy officers and certified (assistant) nurses expressed feeling more in control of daily care tasks and of the process of QI. This, in turn, helped them better comply with quality standards, also after the programme had ended.
  Director: ‘We were constantly having to deal with so many issues within the organization at the same time—it [the PDCA cycle] really helped us gain focus and approach tasks in a step-by-step manner.’
  Expert operational coach: ‘Organizations became more aware of the advantages of a systematic way of working. Without it, mistakes, misunderstandings and noncompliance were much more likely.’

3) *A shift towards adopting well-being and quality of life* as the starting points both for high-quality resident care and for interactions with informal caregivers. Before the programme, care provision was often described in terms of specific tasks and physical health indicators. After the programme, healthcare professionals expressed increased awareness of residents’ wishes, needs and preferences, and reported striving to act accordingly.
  Quality officer: ‘Before D&p, PCC was present in the boardroom, but not on the work floor.’
  Certified nurse: ‘We now pay much more attention to the needs of residents. That makes the work more pleasant for us—and clients notice it, too.’

### The role of expert coaches within D&p

Throughout the interviews, healthcare professionals were questioned about the added value of the expert coaches within the D&p trajectories. Overall, interviewees agreed that the expert coaches had substantially contributed to the improvement process in several ways: 1) by evaluating the situation from an outsider’s perspective, 2) by bringing in experience and expertise, 3) by helping the organization stay committed and focused, and 4) by facilitating communication within teams and between the work floor and management. These factors are described in more depth below.

1. *Outsider’s perspective*
  According to interviewees, both the outsider’s view and independent position of the coaches helped nursing home organizations to ‘analyse the situation more objectively’ and ‘quickly get to the root cause of the problem’. Compared to organization employees, coaches were less hindered by blind spots or fixed patterns. By ‘holding up a mirror’ and confronting staff members when necessary, the expert coaches contributed to ‘a sense of urgency’ within the organization regarding the need for change.

2. *Experience and expertise*
  Interviewees noted the coaches’ experience and expertise vis-à-vis quality standards and change management as ‘helping to translate desired goals into concrete actions’. Without external help, setting up and implementing a concrete action plan covering all eight quality themes would have been challenging for most of the nursing home organizations. During the implementation process, coaches ‘brought in knowledge, practical tools and lessons they had learned from other nursing home organizations, for example: operational coaches engaged with nurses in teams and helped them ‘learn by doing’ by demonstrating how to draft care plans; strategic coaches supported the board and management by serving as a ‘sounding board’ when making strategic decisions with respect to quality of care or conditions for QI.

3. *Commitment and focus*
  The frequent on-site presence of the coaches helped organizations stay committed to their QI plans throughout the 2-year process. Described by interviewees as being ‘important continuous factors’ and ‘beacons in a raging sea’, the coaches provided employees with focus and structure both by repeatedly bringing QI goals and plans to the attention of the board, management and nurses, and by implementing a PDCA cycle on all levels of the organization. This encouraged all stakeholders to ‘stay on track’ and ‘focus on the long-term goals instead of on the disturbance of the day’.

4. *Facilitating communication*
  According to interviewees, the expert coaches facilitated communication both within professional teams, and between management and the work floor. Coaches ‘made people aware of existing friction and troubles’ and helped to ‘open up discussion around things previously left unsaid’. They did so by creating a ‘safe and open environment for conversation’ and by ‘uncovering long-lasting patterns between people’. Greatly valued by participating organizations for their combined ability to ‘bring together policy and practice’, the operational and strategic coaches regularly communicated and engaged with each other: issues on the work floor were therefore signalled more quickly, and new or updated protocols, standards or methods were more readily translated into everyday care routines.

## Discussion

To improve the quality of care in nursing homes with urgent quality issues, the Dutch government’s nationwide support programme—entitled Dignity and pride (D&p)—followed a whole-system approach, targeting not only PCC and resident safety, but also conditional factors such as personnel planning and governance. A key element of the programme was the on-site, tailored support of external expert coaches, whose task was both to guide nursing home organizations through the change process and to ensure the involvement of all necessary internal stakeholders. In this article, we have investigated the effectiveness of the programme for improving PCC and resident safety, and elaborated on the added value of the expert coaches throughout the improvement process.

### Effectiveness of the D&p programme

Confirmed in interviews with healthcare professionals and other stakeholders, the results of this study show significant improvements in PCC and resident safety in almost all 36 participating nursing home organizations. According to interviewees, daily practice most benefited from ‘an improved awareness of quality standards among nurses and staff’, ‘an increased focus on systematic monitoring and quality care improvement’, and ‘a shift towards residents’ well-being and quality of life’. In addition, implementation of the PDCA cycle stimulated nursing home organizations to continue improving their quality of care even after the programme had ended.

It should be noted that the D&p programme only included nursing home organizations with urgent quality issues. Due to this selection bias, results cannot be generalized to all nursing home organizations in the Netherlands [16]. Many of the nursing home organizations participating in D&p were under increased attention of the Health Care Inspectorate (of which 44% under ‘strict supervision’), which undoubtably had a large impact on employees’ sense of urgency and motivation to change. Further research is required to determine to what extent nursing home organizations with milder quality issues may be able to achieve similar improvements.

Complementing the quantitative data gathered on QI, interviewees regarded increased attention for residents’ needs and wishes as one of the greatest benefits of participation in the D&p programme. This result also aligns with the central position of PCC both in the Quality Framework for Nursing Home Care [6, 10] and in other recent European policy documents [1, 2]. It should, however, be kept in mind that a whole-system approach is essential to realizing high-quality care, targeting PCC in conjunction with other quality themes and conditional factors [1, 10].

Although our quantitative QI data are presented as straight lines (Fig. 4), in reality the process of organizational change is usually quite rocky [25]. Multiple (un)foreseen circumstances can impact the course of a change trajectory, e.g., board members leaving the organization, a lack of skilled personnel or severe financial problems. Such extenuating circumstances were especially present for the organizations participating in D&p, highlighting the need for a flexible approach to QI: activities need to be carefully planned, but also easily adapted when unexpected challenges arise [9, 25, 26]. Aware of these potential pitfalls and recent views on QI, the design of the D&p programme left room for improvement plans to be adjusted along the way.

Despite the positive results on PCC and resident safety, to what extent the quality improvements realized within D&p will be maintained over time remains unknown. Especially as COVID-19 presented itself in the Netherlands soon after the D&p programme had been completed, with major implications for society, many nursing home organizations experienced a setback. In all 36 trajectories, internal ownership was a main goal of intervention, strategically pursued by, for example, appointing site champions [8, 16] and involving staff in training and monitoring activities [27, 28]. Internal ownership is regarded as an essential to the success and sustainability of QI [8, 16, 17]. Another strategy with respect to sustainable QI was the use of a systematic monitoring and problem-solving process (PDCA cycle) [8, 16]—implementation of which was indeed highlighted by participating organizations as one of the most valuable benefits of the programme.

### Added value of external expert coaches

Based on the experiences of directors, policy officers and certified (assistant) nurses, the added value of the expert coaches was evaluated qualitatively. Interviewees were consistently positive about the external support they had received, indicating that the expert coaches had contributed to the QI process in several ways. First and foremost, their ability to create a sense of urgency for change was often mentioned—a well-known vital step in organizational change management [29]—strengthened by their abilities to critically reflect on the organization’s situation, to initiate open and honest discussions, and to request the involvement of key stakeholders. The independent perspective provided by their ‘outsider’ status helped them achieve these goals. Moreover, the coaches were credited with stimulating employee commitment, creating focus and structure, facilitating communication across the organization, and providing staff with crucial knowledge and skills [8, 16, 17].

Notable is that several of the positive factors attributed to the influence of the expert coaches pertained to behavioural qualities, such as employees’ sense of urgency, commitment, and open communication. Although such behavioural factors are known to have a large impact on the effectiveness of QI [30], they are also elusive and notoriously difficult to change. According to interviewees, the frequent and long-term presence of the expert coaches, in combination with their independent position, helped to create a climate of openness and trust in which new routines could be shaped and practised [17]. Described as being essential to the realization of high-quality care by the Dutch Quality Framework for Nursing Home Care [6, 10], a safe learning environment within organizations, such as that described by interviewees, is thought to be fundamental to effective QI [31, 32].

Expensive and time-consuming, intensive on-site coaching by external expert coaches may not be feasible in every healthcare setting. By scaling up and spreading best practices [18, 20], stimulating innovation [21], or forming QI collaboratives [22], other QI programmes may be able to provide more-lean approaches to QI support. However, for nursing home organizations such as those that participated in D&p—with urgent quality issues and a variety of complicating factors—more intensive support may be necessary.

### Strengths and limitations

Strengths of this study include the large range (size and location) of participating nursing home organizations, the standardized approach to quantifying change in quality of care according to well-described national standards, and the evaluation of user experiences to complement the quantitative results.

Our study also knows three important limitations. First, because quality of care was evaluated retrospectively, quality indicators were not determined beforehand. This may have led to incomplete reporting on PCC and resident safety. However, as all D&p problem analyses, improvement plans and final evaluations had to be structured according to the national Quality Framework for Nursing Home Care, these written reports appeared to be fairly thorough and complete.

Second, we cannot completely rule out the possibility that the researchers unintentionally showed subjectivity in the tenet scoring. As the quality of PCC and resident safety is shaped upon a complex interplay of factors on both the individual, team and organizational level [33], it cannot easily be captured by objectively measurable indicators [34]. For the tenet scoring, the researchers therefore thoroughly analysed the improvement plans and final evaluation reports, to form a broad picture of the organizations’ dynamics with respect to PCC and resident safety. This document analysis could, however, to some extent have been subject to raters’ interpretation, or the researchers unintentionally could have been affected by exposure to organization’s names, which we were unable to anonymize when scoring quality of care. We therefore objectified the tenet scoring as much as possible by using a standardized scoring form, by having two independent raters assign scores, by randomizing both organization reports, and start and end dates across raters and by performing an external audit to assure transparency of the scoring procedure. Post-hoc analysis revealed no rater bias. Also, the independent external auditors agreed with the assessment procedure and the findings as presented in this study. In addition, in cases where recent inspectorate reports were available for participating organizations, their results were consistent with our findings.

Third, as interviews were held after finalizing the QI trajectory, verbal information relied on the memories of the interviewees, which may have been selective. In addition, given that positive results were obtained in the improvement trajectories and intensive coaching was provided free of charge, interviewees may have felt hesitant to negatively comment on the expert coaches. Despite these uncertainties, the interviews showed a consistent image both of the added value of the expert coaches and of the most significant benefits of the programme.

## Conclusions

The results presented in this study suggest that the D&p programme was associated with improved quality of care in nursing home organizations with urgent quality issues. All nursing home organizations studied showed increased resident safety and person-centred care after completion of the programme, while the expert coaches were perceived as vitally advancing the QI process by stimulating open discussion and critical reflection, creating focus and structure, facilitating communication, and providing critical knowledge and skills. Despite these positive results, on-site coaching such as that found in D&p may not be feasible in every healthcare setting due to time, labour and budget constraints. Nevertheless, elements of D&p, such as the whole-system approach, the use of blended facilitation, and the focus on using the PDCA-cycle should be regarded as valuable ingredients for QI support.

## Data Availability

The datasets generated and/or analysed during the current study are not publicly available due to privacy limitations, but are available from the corresponding author upon reasonable request.

## Abbreviations

QI: Quality Improvement
D&p: Dignity and pride
PCC: Person-Centred Care
